# Dynamic Analysis of the Multi-Lingual S2IR Rumor Propagation Model Under Stochastic Disturbances

**DOI:** 10.3390/e27030217

**Published:** 2025-02-20

**Authors:** Jinling Wang, Jing Liao, Jun-Guo Lu, Jiarong Li, Mei Liu

**Affiliations:** 1 Department of Automation, Shanghai Jiao Tong University, Shanghai 200240, China; jglu@sjtu.edu.cn (J.-G.L.); ljrmath@sjtu.edu.cn (J.L.); 2 School of Mathematics and Statistics, Northwest Normal University, Lanzhou 730070, China; liaojing_00@163.com; 3 School of Mathematics and Statistics, Zhoukou Normal University, Zhoukou 466001, China; 20181013@zknu.cn

**Keywords:** rumor propagation, multi-lingual environment, stochastic disturbances, stochastic optimal control

## Abstract

This paper proposes a multi-lingual S2IR rumor propagation model with white noise disturbances, aiming to study its dynamics and stochastic optimal control strategies. Firstly, a deterministic model is developed within a multi-lingual environment to identify rumor-free and rumor-spreading equilibria and calculate the basic reproduction number $R_{0}$. Secondly, a stochastic model incorporating white noise perturbation is developed, and the uniqueness of its global positive solution is examined. Meanwhile, the asymptotic behaviors of the model’s global solution near the steady states are discussed. Thirdly, the stochastic optimal control is designed to suppress the spread of rumors. Finally, the correctness and validity of the theoretical results are verified through numerical simulation.

## 1. Introduction

Rumor refers to information that emerges and spreads in society without being officially publicly verified. It is characterized by rapid dissemination and a wide reach, disrupting the social dissemination of authoritative voices [1,2]. Particularly in the current Omnimedia era with globalization and the highly developed Internet, the media has achieved in-depth integration with the economy, politics, technology, and so on. This integration has not only changed the form of information dissemination but also expanded the coverage of information audiences. Meanwhile, it plays a role like a magnifying glass in the spread of rumors, bringing serious challenges to social order and public perception [3,4]. In addition, information dissemination crosses language boundaries and is full of uncertainties, and rumors also spread wantonly among different language groups [5]. Therefore, it is of great significance to study the internal mechanism of rumor propagation in online social networks to explore targeted rumor control strategies.

The dynamic behaviors of rumors in the network have become a research hotspot in today’s society. Similar to the spread of infectious diseases, the generation and spread of rumors have also become a social contagion process among users of Online Social Networks (OSNs). The research on rumor spreading started in 1964. Daley and Kendall [6] first proposed the rumor-spreading model. Subsequently, Thompson and Maki [7] considered the more complex interactions between the ignorant and the spreader and proposed the MT model. These rumor-spreading theories have laid an important foundation for subsequent research. Zanette [8] and Moreno et al. [9] studied the dynamics of rumor spreading by using complex networks. Since then, a large number of valuable applied research results have been discovered in the field of complex networks [10,11,12]. In addition, some scholars have committed to studying the nonlinear rumor-spreading rate based on individual differences [13,14]. Currently, in order to make the research on rumor spreading in social networks more in line with reality, many scholars consider other factors in the models, such as the forgetting mechanism [15], the hesitation mechanism [16], government punishment and control [17], the memory effect [18], and so on. They reveal the characteristics of rumor spreading in the OSNs from different perspectives.

Most of the existing literature is limited to the exploration of internal characteristics, and seldom results consider the intervention effect of external information on rumor spreading. In actual rumor spreading scenarios, there are random perturbation situations caused by sudden social events, the alternation of online hotspots, collisions of different cultures, and so on. These real situations make rumor spreading full of randomness and unpredictability. Therefore, it is necessary to introduce random perturbation factors into the model. Jiang et al. [19] established a stochastic SIR model by introducing white noise interference into the whole system, and this noise was proportional to the individuals in the three states. Jia et al. [20] considered mechanisms such as noise and time lag and studied the existence, extinction, and asymptotics of the solutions of the model.

However, the above research work mainly considered the spread of rumors in a single-language environment. Usually, the process of rumor spreading in reality is rather complicated, and there are cases where rumors spread through multiple languages. In a multi-lingual environment, the diffusion of rumors from one language group to another will be affected by language barriers, cultural differences, and so on. There are specific probabilities and conditions for its spreading and transformation, and these mechanisms jointly affect the overall rumor-spreading situation. Therefore, Li et al. [21] established and analyzed the I2S2R rumor-spreading model in complex networks and discussed the dynamics through the mean-field equation. Furthermore, Wang et al. [22] introduced a nonlinear inhibition mechanism into the multi-lingual rumor-spreading model, analyzed the existence and stability of the equilibrium points, as well as the Hopf bifurcation, and designed the optimal control strategy for the model.

The uncertain factors of the network and the multi-lingual environment enable rumors to spread rapidly. If they are not controlled, the spreading range may expand quickly and cause adverse impacts on society. Traditional control methods may not be very effective. However, by introducing the optimal control, corresponding control strategies can be formulated according to the characteristics of rumor spreading, such as releasing rumor-refuting information in a timely manner and strengthening the punishment for rumor spreaders, so as to improve the efficiency of resource utilization and thus reduce the control cost [23,24]. Therefore, we introduce the stochastic optimal control in the process of rumor spreading under stochastic interference to keep rumors within a certain range at the minimum cost.

In order to comprehensively explore the impact of stochastic disturbance on rumor spreading in a multi-lingual environment, we propose a new stochastic S2IR rumor-spreading model. The main work of this paper is shown as follows:

(1) Compared with existing literature [22,25], both stochastic disturbances and the multi-lingual environment are considered to establish a multi-lingual stochastic S2IR rumor-spreading model.

(2) The existence and uniqueness of the global positive solution for the stochastic rumor model, along with the asymptotic behavior of the solution near the steady states, are investigated.

(3) To minimize the number of rumor spreaders at the lowest possible cost and provide a theoretical reference for effectively controlling rumors, the stochastic optimal control strategy is put forward here.

The structure of this paper is shown below. Section 2 establishes the deterministic and its stochastic rumor-spreading model. In Section 3, the uniqueness of its global positive solution and the asymptotic behaviors near the steady-states are obtained. Section 4 proposes a stochastic optimal control model. Section 5 completes the theoretical verification through numerical simulation, and Section 6 presents the conclusions.

## 2. Model Formulation and Preliminaries

### 2.1. Multi-Lingual Deterministic Model

Assume that the total number of users at time *t* is N(t), the population is divided into four categories: ignorants S(t) (users who do not know rumors but are easily infected), spreaders $I_{i}\left(t\right)$ (users who master two languages, and use language *i* to spread rumors, i=1,2), and stiflers R(t) (users who identify rumors and lose interest in spreading them). Specifically, when ignorants come into contact with rumors, they can react in three ways: they can contact spreaders $I_{1}\left(t\right)$ and $I_{2}\left(t\right)$ with the probability of α to become spreaders $I_{1}\left(t\right)$ to spread rumors, or contact spreaders $I_{1}\left(t\right)$ and $I_{2}\left(t\right)$ with the probability of β to become spreaders $I_{2}\left(t\right)$, or simply ignore rumors with the probability of γ to become a stifler R(t) due to lack of interest and inability to know the authenticity of the source of rumors. In addition, some rumor spreaders become stiflers with probability θ due to the forgetting phenomenon of rumors, and others are prevented or reported at the speed of μ as punishment for spreading any harmful rumors. Here, suppose that the new users per unit time do not know the rumor information and the number is constant; that is, the ignorant’s migration rate is Λ. Meanwhile, each type of user exits the platform at a constant rate *d* per unit time.

Then, the deterministic S2IR rumor propagation model is established as follows:

(1) $$\left\{\begin{array}{c} \frac{dS(t)}{dt}=\Lambda -\left(\alpha +\beta \right)\langle k\rangle S\left(t\right)\left(I_{1}\left(t\right)+I_{2}\left(t\right)\right)-\left(d+\gamma \right)S\left(t\right),\\ \frac{dI_{1}\left(t\right)}{dt}=\alpha \langle k\rangle S\left(t\right)\left(I_{1}\left(t\right)+I_{2}\left(t\right)\right)-\left(d+\theta +\mu \right)I_{1}\left(t\right),\\ \frac{dI_{2}\left(t\right)}{dt}=\beta \langle k\rangle S\left(t\right)\left(I_{1}\left(t\right)+I_{2}\left(t\right)\right)-\left(d+\theta +\mu \right)I_{2}\left(t\right),\\ \frac{dR(t)}{dt}=\left(\theta +\mu \right)\left(I_{1}\left(t\right)+I_{2}\left(t\right)\right)+\gamma S\left(t\right)-dR\left(t\right), \end{array}\right.$$

where the initial condition is

$$S\left(0\right)\geq 0,I_{i}\left(0\right)\geq 0,R\left(0\right)\geq 0,i=1,2.$$

Table 1 provides a comprehensive description of the model parameters.

In addition, the discussion of the positive invariant set of model (1) is similar to literature [26], which can be expressed as

$$\Omega =\left\{\left(S\left(t\right),I_{1}\left(t\right),I_{2}\left(t\right),R\left(t\right)\right)\in R_{+}^{4}|S\left(t\right)+I_{1}\left(t\right)+I_{2}\left(t\right)+R\left(t\right)\leq \frac{\Lambda }{d}\right\}.$$

Obviously, the rumor-free equilibrium $E_{0}$ of model (1) is $\left(\frac{\Lambda }{d+\gamma },0,0,\frac{\Lambda \gamma }{d(d+\gamma )}\right)$, and the basic reproduction number $R_{0}$ is $\frac{\left(\alpha +\beta \right)\langle k\rangle \Lambda }{\left(d+\gamma )(d+\theta +\mu \right)}$, which can be obtained by geometric method [27] or next-generation matrix method [28]. In conclusion, $E_{0}$ is stable for $R_{0}<1$, but unstable for $R_{0}>1$.

Next, consider the rumor-spreading equilibrium $E^{*}=\left(S^{*},I_{1}^{*},I_{2}^{*},R^{*}\right)$. Let $\eta =I_{1}^{*}+I_{2}^{*}\geq 0$ and substitute η and $E^{*}$ into model (1), we have

$$S^{*}=\frac{\Lambda }{\left(\alpha +\beta \right)\langle k\rangle \eta +d+\gamma },\;I_{1}^{*}=\frac{\alpha \langle k\rangle \eta S^{*}}{d+\theta +\mu },\;I_{2}^{*}=\frac{\beta \langle k\rangle \eta S^{*}}{d+\theta +\mu }.$$

Through further calculation, one can obtain

$$I_{1}^{*}+I_{2}^{*}=\frac{\left(\alpha +\beta \right)\langle k\rangle \Lambda \eta }{\left(d+\theta +\mu \right)[\left(\alpha +\beta \right)\langle k\rangle \eta +d+\gamma ]}=\eta .$$

Construct the following function

$$f\left(\eta \right)=1-\frac{\left(\alpha +\beta \right)\langle k\rangle \Lambda }{\left(d+\theta +\mu \right)[\left(\alpha +\beta \right)\langle k\rangle \eta +d+\gamma ]}.$$

Since $\dot{f}\left(\eta \right)>0$ and $f\left(\frac{\Lambda }{d}\right)>1-\frac{d}{d+\theta +\mu }>0$, to ensure that f(η)=0 has a unique solution, it is necessary to make f(0)<0, that is

$$1-\frac{\left(\alpha +\beta \right)\langle k\rangle \Lambda }{\left(d+\theta +\mu )(d+\gamma \right)}<0.$$

Hence, when $R_{0}>1$, system (1) has a unique rumor-spreading equilibrium $E^{*}$.

### 2.2. Multi-Lingual Stochastic Model

**Figure 1:**
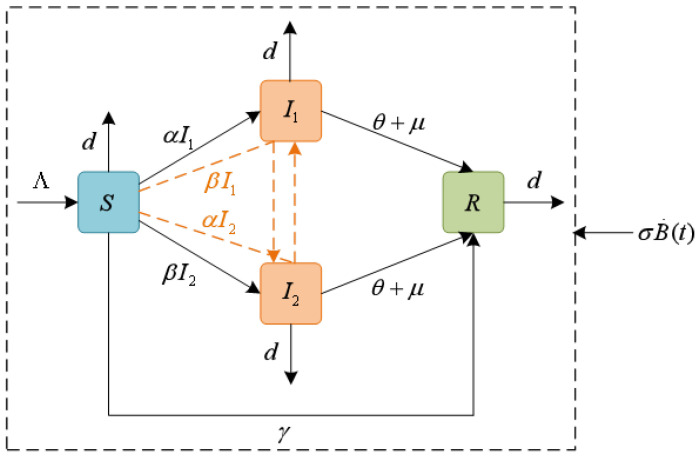
Flow chart of rumor propagation dynamics with the stochastic disturbance.

To quantify the effect of environmental fluctuation on the spread of rumors, based on model (1), we introduce a stochastic disturbance of white noise type in the growth term of the four populations, which is proportional to S(t), $I_{1}\left(t\right)$, $I_{2}\left(t\right)$, R(t). The interactions among various groups of the proposed model are depicted in Figure 1, and the multi-lingual stochastic S2IR rumor propagation model is expressed as

(2) $$\left\{\begin{array}{cc} dS(t)= & \left[\Lambda -\left(\alpha +\beta \right)\langle k\rangle S\left(t\right)\left(I_{1}\left(t\right)+I_{2}\left(t\right)\right)-\left(d+\gamma \right)S\left(t\right)\right]dt\\ & +\sigma_{1}S\left(t\right)dB_{1}\left(t\right),\\ dI_{1}\left(t\right)= & \left[\alpha \langle k\rangle S\left(t\right)\left(I_{1}\left(t\right)+I_{2}\left(t\right)\right)-\left(d+\theta +\mu \right)I_{1}\left(t\right)\right]dt+\sigma_{2}I_{1}\left(t\right)dB_{2}\left(t\right),\\ dI_{2}\left(t\right)= & \left[\beta \langle k\rangle S\left(t\right)\left(I_{1}\left(t\right)+I_{2}\left(t\right)\right)-\left(d+\theta +\mu \right)I_{2}\left(t\right)\right]dt+\sigma_{3}I_{2}\left(t\right)dB_{3}\left(t\right),\\ dR(t)= & \left[\left(\theta +\mu \right)(I_{1}\left(t\right)+I_{2}\left(t\right))+\gamma S\left(t\right)-dR\left(t\right)\right]dt+\sigma_{4}R\left(t\right)dB_{4}\left(t\right), \end{array}\right.$$

where $B_{i}\left(t\right)$ are mutually independent standard Brownian motions defined over a complete probability space $\left(\Omega ,F,F_{t},P\right)$ with a filtration ${\left\{F_{t}\right\}}_{t\geq 0}$, satisfying the usual conditions (that is, it is right continuity and increasing, while $\left\{F_{0}\right\}$ contains all *P*-null sets). Additionally, $\sigma_{i}\left(i=1,2,\ldots ,4\right)$ is the intensity of white noise.

To further analyze the dynamics and control of stochastic S2IR rumor propagation model (2), the following definition is essential to introduce here.

**Lemma** **1** ([29] **(Itô’s formula)**)**.** *Assume x(t) is an n-dimensional Itô’s process on t≥0 and consider the following n-dimensional stochastic differential equations*

(3) dx(t)=f(x(t),t)dt+g(x(t),t)dB(t),

*where $f\in L^{1}\left(R_{+},R^{n}\right)$ and $g\in L^{2}\left(R_{+},R^{n\times m}\right)$(that is, they are Lebesgue integrable on the defined spaces). Let $V\left(x\left(t\right),t\right)\in C^{2,1}\left(R^{n}\times R_{+},R\right)$ be a non-negative continuously differentiable function, then V(x(t),t) is still an Itô’s process. The stochastic differential form is shown as follows:*

(4) $$\begin{array}{c} \;dV(x(t),t)\\ =[V_{t}\left(x\left(t\right),t\right)+V_{x}\left(x\left(t\right),t\right)f\left(x\left(t\right),t\right)+\frac{1}{2}trace\left(g^{T}\left(x\left(t\right),t\right)V_{xx}\left(x\left(t\right),t\right)g\left(x\left(t\right),t\right)\right)]dt\\ \;+V_{x}\left(x\left(t\right),t\right)g\left(x\left(t\right),t\right)dB\left(t\right). \end{array}$$

## 3. Main Results

### 3.1. Existence and Uniqueness of the Positive Global Solution

To study the dynamical properties of the stochastic system, the first concern is whether there exists a positive and global solution. In this section, we will demonstrate that the model (2) has global positive solutions according to the Lyapunov analysis method.

**Theorem** **1.** *For any given initial value $\left(S\left(0\right),I_{1}\left(0\right),I_{2}\left(0\right),R\left(0\right)\right)\in R_{+}^{4}$, the model (2) has a unique global solution $\left(S\left(t\right),I_{1}\left(t\right),I_{2}\left(t\right),R\left(t\right)\right)$ for t≥0 and the solution will remain in $R_{+}^{4}$ with probability one, that is, $\left(S\left(t\right),I_{1}\left(t\right),I_{2}\left(t\right),R\left(t\right)\right)\in R_{+}^{4}$ for all t≥0 almost surely (a.s.).*

**Proof.**  It is obvious that the coefficients of model (2) satisfy local Lipschitz continuity, so for any given initial value $\left(S\left(0\right),I_{1}\left(0\right),I_{2}\left(0\right),R\left(0\right)\right)\in R_{+}^{4}$, the model has a unique local solution $\left(S\left(t\right),I_{1}\left(t\right),I_{2}\left(t\right),R\left(t\right)\right),t\in \left[0,\tau_{e}\right)$, where $\tau_{e}$ is the explosion time. In order to prove the globality of the solution, we only need to prove $\tau_{e}=\infty \;a.s.$

Take a sufficiently large integer $k_{0}>0$ such that S(0), $I_{1}\left(0\right)$, $I_{2}\left(0\right)$, $R\left(0\right)\in \left(\frac{1}{k_{0}},k_{0}\right)$. For any $k\geq k_{0}$, the stopping time $\tau_{k}$ can be defined as

$$\tau_{k}=\inf\left\{t\in \left[0,\tau_{e}\right)\left|S\left(t\right)\notin \left(\frac{1}{k},k\right)\;\mathrm{or}\;I_{1}\left(t\right)\notin \left(\frac{1}{k},k\right)\;\mathrm{or}\;I_{2}\left(t\right)\notin \left(\frac{1}{k},k\right)\;\mathrm{or}\;R\left(t\right)\notin \left(\frac{1}{k},k\right)\right.\right\}.$$

where inf∅=∞ (∅ denotes empty set). According to the definition of stopping time, $\tau_{k}$ is monotonically increasing as k→∞, so we let $\tau_{\infty }=\lim_{k\to \infty }\tau_{k}$ and then $\tau_{\infty }\leq \tau_{e}\;a.s.$ If it is true that $\tau_{\infty }=\infty \;a.s.$ then $\tau_{e}=\infty \;a.s.$ and $\left(S\left(t\right),I_{1}\left(t\right),I_{2}\left(t\right),R\left(t\right)\right)\in R_{+}^{4}\;a.s.,\;t\geq 0$. Suppose that $\tau_{\infty }\neq \infty \;a.s.$, then there exists a pair of constants T>0 and $\varepsilon \in \left(0,1\right)$ such that $P\left\{\tau_{\infty }\leq T\right\}>\varepsilon$. Therefore, there is an integer $k_{1}\geq k_{0}$ such that $P\left\{\tau_{k}\leq T\right\}\geq \varepsilon$ for all $k\geq k_{1}$.

Define a function *V*: $R_{+}^{4}\to \left[0,+\infty \right)$ as

(5) $$V\left(S,I_{1},I_{2},R\right)=S-a-a\ln\frac{S}{a}+I_{1}-1-\ln I_{1}+I_{2}-1-\ln I_{2}+R-1-\ln R,$$

with $a=\frac{d}{\left(\alpha +\beta \right)\langle k\rangle }$. Then, based on Lemma 1, we have

(6) $$\begin{array}{cc} dV(S,I_{1},I_{2},R)= & LV\left(S,I_{1},I_{2},R\right)dt+\left(S-a\right)\sigma_{1}dB_{1}\left(t\right)+\left(I_{1}-1\right)\sigma_{2}dB_{2}\left(t\right)\\ & +\left(I_{2}-1\right)\sigma_{3}dB_{3}\left(t\right)+\left(R-1\right)\sigma_{4}dB_{4}\left(t\right), \end{array}$$

where

$$\begin{array}{cc} & LV(S,I_{1},I_{2},R)\\ = & \;\left(1-\frac{a}{S}\right)\left[\Lambda -\left(\alpha +\beta \right)\langle k\rangle \left(I_{1}+I_{2}\right)S-\left(d+\gamma \right)S\right]+\frac{1}{2}a\sigma_{1}^{2}\\ & +\left(1-\frac{1}{I_{1}}\right)\left[\alpha \langle k\rangle \left(I_{1}+I_{2}\right)S-\left(d+\theta +\mu \right)I_{1}\right]+\frac{1}{2}\sigma_{2}^{2}\\ & +\left(1-\frac{1}{I_{2}}\right)\left[\beta \langle k\rangle \left(I_{1}+I_{2}\right)S-\left(d+\theta +\mu \right)I_{2}\right]+\frac{1}{2}\sigma_{3}^{2}\\ & +\left(1-\frac{1}{R}\right)\left[\left(\theta +\mu \right)\left(I_{1}+I_{2}\right)+\gamma S-dR\right]+\frac{1}{2}\sigma_{4}^{2}. \end{array}$$

Further, one has

$$\begin{array}{cc} & LV(S,I_{1},I_{2},R)\\ = & \;\Lambda -dS-dI_{1}-dI_{2}-dR-\Lambda \frac{a}{S}+a\left(\alpha +\beta \right)\langle k\rangle \left(I_{1}+I_{2}\right)+a\left(d+\gamma \right)\\ & -\frac{\alpha \langle k\rangle \left(I_{1}+I_{2}\right)S}{I_{1}}+2\left(d+\theta +\mu \right)-\frac{\beta \langle k\rangle \left(I_{1}+I_{2}\right)S}{I_{2}}-\frac{\left(\theta +\mu \right)\left(I_{1}+I_{2}\right)}{R}\\ & -\frac{\gamma S}{R}+d+\frac{1}{2}\left(a\sigma_{1}^{2}+\sigma_{2}^{2}+\sigma_{3}^{2}+\sigma_{4}^{2}\right)\\ \leq & \;\Lambda +\left[a\left(\alpha +\beta \right)\langle k\rangle -d\right]\left(I_{1}+I_{2}\right)+a\left(d+\gamma \right)+2\left(d+\theta +\mu \right)+d\;\;\;\\ & +\frac{1}{2}\left(a\sigma_{1}^{2}+\sigma_{2}^{2}+\sigma_{3}^{2}+\sigma_{4}^{2}\right)\\ \leq & \;\Lambda +a\left(d+\gamma \right)+2\left(d+\theta +\mu \right)+d+\frac{1}{2}\left(a\sigma_{1}^{2}+\sigma_{2}^{2}+\sigma_{3}^{2}+\sigma_{4}^{2}\right)\\ \overset{▵}{=} & \;K. \end{array}$$

Next, integrating Equation (6) from 0 to $\tau_{k}\wedge T\overset{▵}{=}\min\left\{\tau_{k},T\right\}$ and taking expectation on both sides, we obtain

(7) $$\begin{array}{cc} \; & EV(S\left(\tau_{k}\wedge T\right),I_{1}\left(\tau_{k}\wedge T\right),I_{2}\left(\tau_{k}\wedge T\right),R\left(\tau_{k}\wedge T\right))\\ \leq & \;V\left(S\left(0\right),I_{1}\left(0\right),I_{2}\left(0\right),R\left(0\right)\right)+KE\left(\tau_{k}\wedge T\right)\\ \leq & \;V(S\left(0\right),I_{1}\left(0\right),I_{2}\left(0\right),R\left(0\right))+KT. \end{array}$$

Define $\Omega_{k}=\left\{\tau_{k}\leq T\right\}$ for $k\geq k_{1}$, then it yields $P(\Omega_{k})\geq \varepsilon$. Therefore, for $\forall \omega \in \Omega_{k}$, there exist $S(\tau_{k},\omega )$, $I_{1}\left(\tau_{k},\omega \right)$, $I_{2}\left(\tau_{k},\omega \right)$, $R(\tau_{k},\omega )$ which equal either *k* or $\frac{1}{k}$. Consequently, it follows that

(8) $$\begin{array}{cc} \; & V(S\left(\tau_{k},\omega \right),I_{1}\left(\tau_{k},\omega \right),I_{2}\left(\tau_{k},\omega \right),R\left(\tau_{k},\omega \right))\\ \geq & \left(k-a-a\ln\frac{k}{a}\right)\wedge \left(\frac{1}{k}-a+a\ln ka\right)\wedge \left(k-1-\ln k\right)\wedge \left(\frac{1}{k}-1+\ln k\right). \end{array}$$

Combining Equation (7) with Equation (8), we have

$$\begin{array}{cc} & V(S\left(0\right),I_{1}\left(0\right),I_{2}\left(0\right),R\left(0\right))+KT\\ \geq & E\left[I_{\Omega_{k}}\left(\omega \right)V\left(S\left(\tau_{k},\omega \right),I_{1}\left(\tau_{k},\omega \right),I_{2}\left(\tau_{k},\omega \right),R\left(\tau_{k},\omega \right)\right)\right]\\ \geq & \varepsilon \left[\left(k-a-a\ln\frac{k}{a}\right)\wedge \left(\frac{1}{k}-a+a\ln ka\right)\wedge \left(k-1-\ln k\right)\wedge \left(\frac{1}{k}-1+\ln k\right)\right], \end{array}$$

where $I_{\Omega_{k}}\left(\omega \right)$ is the indicator function of $\Omega_{k}$. Let k→∞, we find that ∞>V(S(0),$I_{1}\left(0\right),I_{2}\left(0\right),R\left(0\right))+KT=\infty$ indicates a contradiction. So we finally get $\tau_{\infty }=\infty \;a.s.$ □

### 3.2. Asymptotic Behaviors of Stochastic Model (2) Around $E_{0}$

If $R_{0}<1$, the rumor-free equilibrium $E_{0}=\left(\frac{\Lambda }{d+\gamma },0,0,\frac{\Lambda \gamma }{d(d+\gamma )}\right)$ of the deterministic model (1) has local asymptotic stability. However, due to the stochastic disturbance, the solutions of stochastic model (2) cannot converge to $E_{0}$. In this section, we shall investigate the asymptotic behaviors of the stochastic model (2) around $E_{0}$.

**Theorem** **2.** *If $R_{0}<1$, then for any initial value $\left(S\left(0\right),I_{1}\left(0\right),I_{2}\left(0\right),R\left(0\right)\right)\in R_{+}^{4}$, the solution $\left(S\left(t\right),I_{1}\left(t\right),I_{2}\left(t\right),R\left(t\right)\right)$ of model (2) has the following property*

(9) $$\begin{array}{cc} \lim_{t\to \infty }\sup & \frac{1}{t}E\int_{0}^{t}\left[{\left(S\left(s\right)-\frac{\Lambda }{d+\gamma }\right)}^{2}+I_{1}^{2}\left(s\right)+I_{2}^{2}\left(s\right)+{\left(R\left(s\right)-\frac{\Lambda \gamma }{d(d+\gamma )}\right)}^{2}\right]ds\\ \leq & \;\frac{2\Lambda^{2}\left[\sigma_{1}^{2}{\left(d+\gamma \right)}^{2}+\sigma_{4}^{2}\gamma^{2}\right]}{d^{2}{\left(d+\gamma \right)}^{2}Q_{1}}, \end{array}$$

*where $Q_{1}=\min\left\{2\left(d-\sigma_{1}^{2}\right),2d-\sigma_{2}^{2},2d-\sigma_{3}^{2},2\left(d-\sigma_{4}^{2}\right)\right\}$ and $\sigma_{1}^{2}<d$, $\sigma_{2}^{2}<2d$, $\sigma_{3}^{2}<2d$, $\sigma_{4}^{2}<d$. (It illustrates that the solution randomly oscillates near the rumor-free equilibrium $E_{0}$ in the sense of time average).*

**Proof.**  Let $X=S\left(t\right)-\frac{\Lambda }{d+\gamma }$, $Y_{1}=I_{1}\left(t\right)$, $Y_{2}=I_{2}\left(t\right)$, $Z=R\left(t\right)-\frac{\Lambda \gamma }{d(d+\gamma )}$, model (2) is transformed into the following form

$$\left\{\begin{array}{cc} dX(t)= & \left[\Lambda -\left(\alpha +\beta \right)\langle k\rangle \left(X\left(t\right)+\frac{\Lambda }{d+\gamma }\right)\left(Y_{1}\left(t\right)+Y_{2}\left(t\right)\right)\right.\\ & \left.-\left(d+\gamma \right)(X\left(t\right)+\frac{\Lambda }{d+\gamma })\right]dt+\sigma_{1}\left(X\left(t\right)+\frac{\Lambda }{d+\gamma }\right)dB_{1}\left(t\right),\\ dY_{1}\left(t\right)= & \left[\alpha \langle k\rangle \left(X\left(t\right)+\frac{\Lambda }{d+\gamma }\right)\left(Y_{1}\left(t\right)+Y_{2}\left(t\right)\right)-\left(d+\theta +\mu \right)Y_{1}\left(t\right)\right]dt\\ & +\sigma_{2}Y_{1}\left(t\right)dB_{2}\left(t\right),\\ dY_{2}\left(t\right)= & \left[\beta \langle k\rangle \left(X\left(t\right)+\frac{\Lambda }{d+\gamma }\right)\left(Y_{1}\left(t\right)+Y_{2}\left(t\right)\right)-\left(d+\theta +\mu \right)Y_{2}\left(t\right)\right]dt\\ & +\sigma_{3}Y_{2}\left(t\right)dB_{3}\left(t\right),\\ dZ(t)= & \left[\left(\theta +\mu \right)\left(Y_{1}\left(t\right)+Y_{2}\left(t\right)\right)+\gamma \left(X\left(t\right)+\frac{\Lambda }{d+\gamma }\right)\right.\\ & \left.-d\left(Z\left(t\right)+\frac{\Lambda \gamma }{d(d+\gamma )}\right)\right]dt+\sigma_{4}\left(Z\left(t\right)+\frac{\Lambda \gamma }{d(d+\gamma )}\right)dB_{4}\left(t\right). \end{array}\right.$$

Consider a Lyapunov function *V* as follows:

$$V\left(X,Y_{1},Y_{2},Z\right)={\left(X+Y_{1}+Y_{2}+Z\right)}^{2}+c_{1}\left(Y_{1}+Y_{2}\right),$$

where $c_{1}=\frac{4d}{\left(\alpha +\beta \right)\langle k\rangle }$ is a positive constant. According to Itô’s formula, we have

(10) $$\begin{array}{cc} dV= & \;LVdt+2\sigma_{1}\left(X+\frac{\Lambda }{d+\gamma }\right)\left(X+Y_{1}+Y_{2}+Z\right)dB_{1}\left(t\right)\\ \; & +\sigma_{2}Y_{1}\left[2\left(X+Y_{1}+Y_{2}+Z\right)+c_{1}\right]dB_{2}\left(t\right)\\ \; & +\sigma_{3}Y_{2}\left[2\left(X+Y_{1}+Y_{2}+Z\right)+c_{1}\right]dB_{3}\left(t\right)\\ \; & +2\sigma_{4}\left(Z+\frac{\Lambda \gamma }{d(d+\gamma )}\right)\left(X+Y_{1}+Y_{2}+Z\right)dB_{4}\left(t\right), \end{array}$$

where

$$\begin{array}{cc} LV= & \;2\left(X+Y_{1}+Y_{2}+Z\right)\left(-dX-dY_{1}-dY_{2}-dZ-\frac{\Lambda \gamma }{d(d+\gamma )}\right)\\ \; & +c_{1}\left[\left(\alpha +\beta \right)\langle k\rangle \left(X+\frac{\Lambda }{d+\gamma }\right)\left(Y_{1}+Y_{2}\right)-\left(d+\theta +\mu \right)\left(Y_{1}+Y_{2}\right)\right]\\ \; & +\sigma_{1}^{2}{\left(X+\frac{\Lambda }{d+\gamma }\right)}^{2}+\sigma_{2}^{2}Y_{1}^{2}+\sigma_{3}^{2}Y_{2}^{2}+\sigma_{4}^{2}{\left(Z+\frac{\Lambda \gamma }{d(d+\gamma )}\right)}^{2}\\ \leq & \;\left(-2d+2\sigma_{1}^{2}\right)X^{2}+\left(-2d+\sigma_{2}^{2}\right)Y_{1}^{2}+\left(-2d+\sigma_{3}^{2}\right)Y_{2}^{2}+\left(-2d+2\sigma_{4}^{2}\right)Z^{2}\\ \; & +\left[-4d+c_{1}\left(\alpha +\beta \right)\langle k\rangle \right]X\left(Y_{1}+Y_{2}\right)+c_{1}\left(d+\theta +\mu \right)\left(R_{0}-1\right)\left(Y_{1}+Y_{2}\right)\\ \; & +2\sigma_{1}^{2}\frac{\Lambda^{2}}{d^{2}}+2\sigma_{4}^{2}\frac{\Lambda^{2}\gamma^{2}}{d^{2}{\left(d+\gamma \right)}^{2}}. \end{array}$$

Next, since $c_{1}=\frac{4d}{\left(\alpha +\beta \right)\langle k\rangle }$, then $-4d+c_{1}\left(\alpha +\beta \right)\langle k\rangle =0$. In addition, due to $R_{0}<1$, we can further obtain

$$\begin{array}{cc} LV\leq & \;\left(-2d+2\sigma_{1}^{2}\right)X^{2}+\left(-2d+\sigma_{2}^{2}\right)Y_{1}^{2}+\left(-2d+\sigma_{3}^{2}\right)Y_{2}^{2}+\left(-2d+2\sigma_{4}^{2}\right)Z^{2}\\ & +\frac{2\Lambda^{2}\left[\sigma_{1}^{2}{\left(d+\gamma \right)}^{2}+\sigma_{4}^{2}\gamma^{2}\right]}{d^{2}{\left(d+\gamma \right)}^{2}}. \end{array}$$

Integrating Equation (10) from 0 to *t* and taking expectation, we have

$$\begin{array}{cc} 0\leq & \;E\left[V(X,Y_{1},Y_{2},Z)\right]\leq V\left(X\left(0\right),Y_{1}\left(0\right),Y_{2}\left(0\right),Z\left(0\right)\right)\\ & -Q_{1}E\int_{0}^{t}\left[X^{2}\left(s\right)+Y_{1}^{2}\left(s\right)+Y_{2}^{2}\left(s\right)+R^{2}\left(s\right)\right]ds+\frac{2\Lambda^{2}\left[\sigma_{1}^{2}{\left(d+\gamma \right)}^{2}+\sigma_{4}^{2}\gamma^{2}\right]}{d^{2}{\left(d+\gamma \right)}^{2}}t, \end{array}$$

where $Q_{1}=\min\left\{2\left(d-\sigma_{1}^{2}\right),2d-\sigma_{2}^{2},2d-\sigma_{3}^{2},2\left(d-\sigma_{4}^{2}\right)\right\}$. Therefore, one has

$$\begin{array}{cc} \lim_{t\to \infty }\sup & \frac{1}{t}E\int_{0}^{t}\left[{\left(S\left(s\right)-\frac{\Lambda }{d+\gamma }\right)}^{2}+I_{1}^{2}\left(s\right)+I_{2}^{2}\left(s\right)+{\left(R\left(s\right)-\frac{\Lambda \gamma }{d(d+\gamma )}\right)}^{2}\right]ds\\ \leq & \;\frac{2\Lambda^{2}\left[\sigma_{1}^{2}{\left(d+\gamma \right)}^{2}+\sigma_{4}^{2}\gamma^{2}\right]}{d^{2}{\left(d+\gamma \right)}^{2}Q_{1}}. \end{array}$$

□

### 3.3. Asymptotic Behaviors of Stochastic Model (2) Around $E^{*}$

If $R_{0}>1$, then the deterministic model (1) has a rumor-spreading equilibrium $E^{*}=\left(S^{*},I_{1}^{*},I_{2}^{*},R^{*}\right)$. Here, we study the asymptotic behaviors of the global solution $\left(S\left(t\right),I_{1}\left(t\right),I_{2}\left(t\right),R\left(t\right)\right)$ of the stochastic model (2) around the rumor-spreading equilibrium $E^{*}$.

**Theorem** **3.** *If $R_{0}>1$, then for any initial value $\left(S\left(0\right),I_{1}\left(0\right),I_{2}\left(0\right),R\left(0\right)\right)\in R_{+}^{4}$, the solution $\left(S\left(t\right),I_{1}\left(t\right),I_{2}\left(t\right),R\left(t\right)\right)$ of system (2) satisfying*

$$\begin{array}{cc} \; & \lim_{t\to \infty }\sup\frac{1}{t}E\int_{0}^{t}{\left[S\left(s\right)-\frac{2d+\gamma }{2d+\gamma -\sigma_{1}^{2}}S^{*}\right]}^{2}+{\left[I_{1}\left(s\right)-\frac{2d}{2d-\sigma_{2}^{2}}I_{1}^{*}\right]}^{2} \end{array}$$

$$\begin{array}{cc} \; & +{\left[I_{2}\left(s\right)-\frac{2d}{2d-\sigma_{3}^{2}}I_{2}^{*}\right]}^{2}+{\left[R\left(s\right)-\frac{\left(4d^{2}+2d\left(\theta +\mu \right)\right)R^{*}}{4d^{2}+2d\left(\theta +\mu \right)-\left(\theta +\mu +4d\right)\sigma_{4}^{2}}\right]}^{2}ds\\ \leq & \;\frac{2P}{Q_{2}}+\frac{8d^{2}I_{2}^{*2}}{{\left(2d-\sigma_{2}^{2}\right)}^{2}}+\frac{8d^{2}I_{1}^{*2}}{{\left(2d-\sigma_{3}^{2}\right)}^{2}}, \end{array}$$

*where $\sigma_{1}^{2}<2d+\gamma$, $\sigma_{2}^{2}<2d$, $\sigma_{2}^{2}<2d$, $\left(\theta +\mu +4d\right)\sigma_{4}^{2}<4d^{2}+2d\left(\theta +\mu \right)$, and*

$$\begin{array}{cc} Q_{2} & =\min\left\{2d+\gamma -\sigma_{1}^{2},d-\frac{\sigma_{2}^{2}}{2},d-\frac{\sigma_{3}^{2}}{2},\frac{2d^{2}+d\left(\theta +\mu \right)}{\theta +\mu }-\frac{\theta +\mu +4d}{2(\theta +\mu )}\sigma_{4}^{2}\right\},\\ P & =\frac{\left(2d+\gamma \right)\sigma_{1}^{2}}{2d+\gamma -\sigma_{1}^{2}}S^{*2}+\frac{2d^{2}I_{2}^{*2}+4d^{2}I_{1}^{*}I_{2}^{*}+d\sigma_{2}^{2}I_{1}^{*2}}{2d-\sigma_{2}^{2}}+\frac{2d^{2}I_{1}^{*2}+4d^{2}I_{1}^{*}I_{2}^{*}+d\sigma_{3}^{2}I_{2}^{*2}}{2d-\sigma_{3}^{2}}\\ & \;+\frac{\left[2d^{2}+d\left(\theta +\mu \right)\right]\left(\theta +\mu +4d\right)\sigma_{4}^{2}}{\left(\theta +\mu \right)\left[4d^{2}+2d\left(\theta +\mu \right)-\left(\theta +\mu +4d\right)\sigma_{4}^{2}\right]}R^{*2}+\frac{2d+\left(\alpha +\beta \right)\langle k\rangle S^{*}}{2\left(\alpha +\beta \right)\langle k\rangle }\\ & \;\times \left(I_{1}^{*}+I_{2}^{*}\right)\left(\sigma_{2}^{2}+\sigma_{3}^{2}\right). \end{array}$$

It illustrates that the solution randomly oscillates near a point related to $E^{*}$ in the sense of time average.

**Proof.**  Consider the following function:

$$V\left(S,I_{1},I_{2},R\right)=V_{1}+c_{2}V_{2}+c_{3}V_{3},$$

where $c_{2}=\frac{2d+\left(\alpha +\beta \right)\langle k\rangle S^{*}}{\left(\alpha +\beta \right)\langle k\rangle }$, $c_{3}=\frac{2d}{\theta +\mu }$, θ+μ>γ, and

$$\begin{array}{c} V_{1}=\frac{1}{2}\left[{\left(S-S^{*}+I_{1}-I_{1}^{*}+I_{2}-I_{2}^{*}+R-R^{*}\right)}^{2}+{\left(S-S^{*}\right)}^{2}\right],\\ V_{2}=\left(I_{1}+I_{2}\right)-\left(I_{1}^{*}+I_{2}^{*}\right)-\left(I_{1}^{*}+I_{2}^{*}\right)\ln\frac{I_{1}+I_{2}}{I_{1}^{*}+I_{2}^{*}},\;V_{3}=\frac{1}{2}{\left(R-R^{*}\right)}^{2}. \end{array}$$

Applying Ito’s formula, we obtain

$$\begin{array}{cc} LV_{1}= & \;\left(S-S^{*}+I_{1}-I_{1}^{*}+I_{2}-I_{2}^{*}+R-R^{*}\right)\left(\Lambda -dS-dI_{1}-dI_{1}-dR\right)\\ & +\left(S-S^{*}\right)\left[\Lambda -\left(\alpha +\beta \right)\langle k\rangle S\left(I_{1}+I_{2}\right)-\left(d+\gamma \right)S\right]\\ & +\frac{1}{2}\left(2\sigma_{1}^{2}S^{2}+\sigma_{2}^{2}I_{1}^{2}+\sigma_{3}^{2}I_{2}^{2}+\sigma_{4}^{2}R^{2}\right)\\ \leq & -\left(2d+\gamma \right){\left(S-S^{*}\right)}^{2}-d{\left(I_{1}-I_{1}^{*}\right)}^{2}-d{\left(I_{2}-I_{2}^{*}\right)}^{2}-d{\left(R-R^{*}\right)}^{2}\\ & -2d\left(S-S^{*}\right)\left(R-R^{*}\right)-2d\left(I_{1}-I_{1}^{*}\right)\left(I_{2}-I_{2}^{*}\right)-2d\left(I_{1}-I_{1}^{*}\right)\left(R-R^{*}\right)\\ & -2d\left(I_{2}-I_{2}^{*}\right)\left(R-R^{*}\right)-\left[2d+\left(\alpha +\beta \right)\langle k\rangle S^{*}\right]\left(S-S^{*}\right)\left(I_{1}-I_{1}^{*}\right)\\ & -\left[2d+\left(\alpha +\beta \right)\langle k\rangle S^{*}\right]\left(S-S^{*}\right)\left(I_{2}-I_{2}^{*}\right)+\sigma_{1}^{2}S^{2}+\frac{1}{2}\sigma_{2}^{2}I_{1}^{2}+\frac{1}{2}\sigma_{3}^{2}I_{2}^{2}\\ & +\frac{1}{2}\sigma_{4}^{2}R^{2}, \end{array}$$

$$\begin{array}{cc} LV_{2}= & \left(1-\frac{I_{1}^{*}+I_{2}^{*}}{I_{1}+I_{2}}\right)\left[\left(\alpha +\beta \right)\langle k\rangle S\left(I_{1}+I_{2}\right)-\left(d+\theta +\mu \right)\left(I_{1}+I_{2}\right)\right]\\ & +\frac{1}{2}\frac{I_{1}^{*}+I_{2}^{*}}{{\left(I_{1}+I_{2}\right)}^{2}}I_{1}^{2}\sigma_{2}^{2}+\frac{1}{2}\frac{I_{1}^{*}+I_{2}^{*}}{{\left(I_{1}+I_{2}\right)}^{2}}I_{2}^{2}\sigma_{3}^{2}\\ \leq & \;\left(I_{1}-I_{1}^{*}+I_{2}-I_{2}^{*}\right)\left(\alpha +\beta \right)\langle k\rangle \left(S-S^{*}\right)+\frac{1}{2}\left(I_{1}^{*}+I_{2}^{*}\right)\left(\sigma_{2}^{2}+\sigma_{3}^{2}\right),\\ LV_{3}= & \;\left(R-R^{*}\right)\left[\left(\theta +\mu \right)\left(I_{1}+I_{2}\right)+\gamma S-dR\right]+\frac{1}{2}\sigma_{4}^{2}R^{2}\\ = & -d{\left(R-R^{*}\right)}^{2}+\left(\theta +\mu \right)\left(I_{1}-I_{1}^{*}\right)\left(R-R^{*}\right)+\left(\theta +\mu \right)\left(I_{2}-I_{2}^{*}\right)\left(R-R^{*}\right)\\ & +\gamma \left(S-S^{*}\right)\left(R-R^{*}\right)+\frac{1}{2}\sigma_{4}^{2}R^{2}. \end{array}$$

Then, we further have

(11) $$\begin{array}{cc} LV\leq & -\left(2d+\gamma \right){\left(S-S^{*}\right)}^{2}+d{\left(I_{1}-I_{1}^{*}\right)}^{2}+d{\left(I_{2}-I_{2}^{*}\right)}^{2}\\ \; & -d\left(c_{3}+1\right){\left(R-R^{*}\right)}^{2}+\left(-2d+c_{3}\gamma \right)\left(S-S^{*}\right)\left(R-R^{*}\right)+2dI_{1}I_{2}^{*}\\ \; & +2dI_{1}^{*}I_{2}+\left[-2d+c_{3}\left(\theta +\mu \right)\right]\left[\left(I_{1}-I_{1}^{*}\right)\left(R-R^{*}\right)+\left(I_{2}-I_{2}^{*}\right)\left(R-R^{*}\right)\right]\\ \; & +\left[-2d+\left(\alpha +\beta \right)\langle k\rangle \left(c_{2}-S^{*}\right)\right]\left[\left(S-S^{*}\right)\left(I_{1}-I_{1}^{*}\right)+\left(S-S^{*}\right)\left(I_{2}-I_{2}^{*}\right)\right]\\ \; & +\sigma_{1}^{2}S^{2}+\frac{1}{2}\sigma_{2}^{2}I_{1}^{2}+\frac{1}{2}\sigma_{3}^{2}I_{2}^{2}+\left(\frac{1}{2}+c_{3}\right)\sigma_{4}^{2}R^{2}+\frac{1}{2}c_{2}\left(I_{1}^{*}+I_{2}^{*}\right)\left(\sigma_{2}^{2}+\sigma_{3}^{2}\right). \end{array}$$

Since $c_{2}=\frac{2d+\left(\alpha +\beta \right)\langle k\rangle S^{*}}{\left(\alpha +\beta \right)\langle k\rangle }$, $c_{3}=\frac{2d}{\theta +\mu }$ and θ+μ>γ, Equation (11) can be transformed into the following form

$$\begin{array}{cc} LV\leq & -\left(2d+\gamma -\sigma_{1}^{2}\right){\left(S-\frac{2d+\gamma }{2d+\gamma -\sigma_{1}^{2}}S^{*}\right)}^{2}+\frac{\left(2d+\gamma \right)\sigma_{1}^{2}}{2d+\gamma -\sigma_{1}^{2}}S^{*2}-\left(d-\frac{\sigma_{2}^{2}}{2}\right)\\ & \times {\left[I_{1}-\frac{2d}{2d-\sigma_{2}^{2}}\left(I_{1}^{*}+I_{2}^{*}\right)\right]}^{2}-\left(d-\frac{\sigma_{3}^{2}}{2}\right){\left[I_{2}-\frac{2d}{2d-\sigma_{3}^{2}}\left(I_{2}^{*}+I_{1}^{*}\right)\right]}^{2}\\ & +\frac{2d^{2}I_{2}^{*2}+4d^{2}I_{1}^{*}I_{2}^{*}+d\sigma_{2}^{2}I_{1}^{*2}}{2d-\sigma_{2}^{2}}+\frac{2d^{2}I_{1}^{*2}+4d^{2}I_{1}^{*}I_{2}^{*}+d\sigma_{3}^{2}I_{2}^{*2}}{2d-\sigma_{3}^{2}}\\ & -\left[\frac{2d^{2}+d\left(\theta +\mu \right)}{\theta +\mu }-\frac{\left(\theta +\mu +4d\right)\sigma_{4}^{2}}{2(\theta +\mu )}\right]{\left[R-\frac{\left(4d^{2}+2d\left(\theta +\mu \right)\right)R^{*}}{4d^{2}+2d\left(\theta +\mu \right)-\left(\theta +\mu +4d\right)\sigma_{4}^{2}}\right]}^{2}\\ & +\frac{\left[2d^{2}+d\left(\theta +\mu \right)\right]\left(\theta +\mu +4d\right)\sigma_{4}^{2}}{\left(\theta +\mu \right)\left[4d^{2}+2d\left(\theta +\mu \right)-\left(\theta +\mu +4d\right)\sigma_{4}^{2}\right]}R^{*2}+\frac{2d+\left(\alpha +\beta \right)\langle k\rangle S^{*}}{2\left(\alpha +\beta \right)\langle k\rangle }\\ & \times \left(I_{1}^{*}+I_{2}^{*}\right)\left(\sigma_{2}^{2}+\sigma_{3}^{2}\right). \end{array}$$

Next, let $Q_{2}=\min\left\{2d+\gamma -\sigma_{1}^{2},d-\frac{\sigma_{2}^{2}}{2},d-\frac{\sigma_{3}^{2}}{2},\frac{2d^{2}+d\left(\theta +\mu \right)}{\theta +\mu }-\frac{\theta +\mu +4d}{2(\theta +\mu )}\sigma_{4}^{2}\right\}$, and

$$\begin{array}{c} P=\frac{\left(2d+\gamma \right)\sigma_{1}^{2}}{2d+\gamma -\sigma_{1}^{2}}S^{*2}+\frac{2d^{2}I_{2}^{*2}+4d^{2}I_{1}^{*}I_{2}^{*}+d\sigma_{2}^{2}I_{1}^{*2}}{2d-\sigma_{2}^{2}}+\frac{2d^{2}I_{1}^{*2}+4d^{2}I_{1}^{*}I_{2}^{*}+d\sigma_{3}^{2}I_{2}^{*2}}{2d-\sigma_{3}^{2}} \end{array}$$

$$\begin{array}{cc} & +\frac{\left[2d^{2}+d\left(\theta +\mu \right)\right]\left(\theta +\mu +4d\right)\sigma_{4}^{2}}{\left(\theta +\mu \right)\left[4d^{2}+2d\left(\theta +\mu \right)-\left(\theta +\mu +4d\right)\sigma_{4}^{2}\right]}R^{*2}+\frac{2d+\left(\alpha +\beta \right)\langle k\rangle S^{*}}{2\left(\alpha +\beta \right)\langle k\rangle }\\ & \times \left(I_{1}^{*}+I_{2}^{*}\right)\left(\sigma_{2}^{2}+\sigma_{3}^{2}\right). \end{array}$$

Similar to the discussion of Theorem 2, we obtain

$$\begin{array}{cc} 0\leq & \;E\left[V(S,I_{1},I_{2},R)\right]\leq V\left(S\left(0\right),I_{1}\left(0\right),I_{2}\left(0\right),R\left(0\right)\right)+Pt\\ & -Q_{2}E\int_{0}^{t}{\left[S\left(s\right)-\frac{2d+\gamma }{2d+\gamma -\sigma_{1}^{2}}S^{*}\right]}^{2}+{\left[I_{1}\left(s\right)-\frac{2d}{2d-\sigma_{2}^{2}}\left(I_{1}^{*}+I_{2}^{*}\right)\right]}^{2}\\ & +{\left[I_{2}\left(s\right)-\frac{2d}{2d-\sigma_{3}^{2}}\left(I_{2}^{*}+I_{1}^{*}\right)\right]}^{2}+{\left[R\left(s\right)-\frac{\left(4d^{2}+2d\left(\theta +\mu \right)\right)R^{*}}{4d^{2}+2d\left(\theta +\mu \right)-\left(\theta +\mu +4d\right)\sigma_{4}^{2}}\right]}^{2}ds. \end{array}$$

Finally, the following formula is obtained

$$\begin{array}{cc} \lim_{t\to \infty }\sup & \frac{1}{t}E\int_{0}^{t}{\left[S\left(s\right)-\frac{2d+\gamma }{2d+\gamma -\sigma_{1}^{2}}S^{*}\right]}^{2}+{\left[I_{1}\left(s\right)-\frac{2d}{2d-\sigma_{2}^{2}}I_{1}^{*}\right]}^{2}\\ & +{\left[I_{2}\left(s\right)-\frac{2d}{2d-\sigma_{3}^{2}}I_{2}^{*}\right]}^{2}+{\left[R\left(s\right)-\frac{\left(4d^{2}+2d\left(\theta +\mu \right)\right)R^{*}}{4d^{2}+2d\left(\theta +\mu \right)-\left(\theta +\mu +4d\right)\sigma_{4}^{2}}\right]}^{2}ds\\ \leq & \;\frac{2P}{Q_{2}}+\frac{8d^{2}I_{2}^{*2}}{{\left(2d-\sigma_{2}^{2}\right)}^{2}}+\frac{8d^{2}I_{1}^{*2}}{{\left(2d-\sigma_{3}^{2}\right)}^{2}}. \end{array}$$

□

**Remark** **1.** Theorems 2 and 3 show that the solution of model (2) vibrates randomly near the rumor-free and the rumor-spreading equilibria. The amplitude of the vibration near the rumor-free equilibrium is related to the size of $\sigma_{i}^{2},i=1,2,\ldots ,4$. According to Formula (9), it can be seen that the vibration amplitude becomes smaller as the value of $\sigma_{i}^{2}$ decreases. Nevertheless, the vibration amplitude near the rumor-spreading equilibrium is associated with $\sigma_{i}^{2}$ and $E^{*}$. The smaller $\sigma_{i}^{2}$ is, the closer the point ${\bar{E}}^{*}=\left(\frac{2d+\gamma }{2d+\gamma -\sigma_{1}^{2}}S^{*},\frac{2d}{2d-\sigma_{2}^{2}}I_{1}^{*},\frac{2d}{2d-\sigma_{3}^{2}}I_{2}^{*},\frac{4d^{2}+2d\left(\theta +\mu \right)}{4d^{2}+2d\left(\theta +\mu \right)-\left(\theta +\mu +4d\right)\sigma_{4}^{2}}R^{*}\right)$ is to $E^{*}$, and consequently, the smaller the amplitude of vibration will be.

## 4. Stochastic Optimal Control

In this section, we investigate the optimal control problem of the stochastic model and put forward a theoretical method for effectively controlling and dealing with the spread of rumors. Its most significant utility lies in maintaining a balance between minimizing expenditures and curbing the spread of rumors. We present two control variables, $u_{1}\left(t\right)$ and $u_{2}\left(t\right)$, which respectively represent the intensities of external control measures for the spreaders $I_{1}\left(t\right)$ and $I_{2}\left(t\right)$. Our goal is to minimize the number of spreaders and maximize the number of stifler individuals by using the smallest possible values of $u_{1}\left(t\right)$ and $u_{2}\left(t\right)$. Consequently, the stochastic model with control measures can be expressed as

(12) $$\left\{\begin{array}{cc} dS(t)= & \left[\Lambda -\left(\alpha +\beta \right)\langle k\rangle S\left(t\right)\left(I_{1}\left(t\right)+I_{2}\left(t\right)\right)-\left(d+\gamma \right)S\left(t\right)\right]dt\\ & +\sigma_{1}S\left(t\right)dB_{1}\left(t\right),\\ dI_{1}\left(t\right)= & \left[\alpha \langle k\rangle S\left(t\right)\left(I_{1}\left(t\right)+I_{2}\left(t\right)\right)-\left(d+\theta +\mu +u_{1}\left(t\right)\right)I_{1}\left(t\right)\right]dt\\ & +\sigma_{2}I_{1}\left(t\right)dB_{2}\left(t\right),\\ dI_{2}\left(t\right)= & \left[\beta \langle k\rangle S\left(t\right)\left(I_{1}\left(t\right)+I_{2}\left(t\right)\right)-\left(d+\theta +\mu +u_{2}\left(t\right)\right)I_{2}\left(t\right)\right]dt\\ & +\sigma_{3}I_{2}\left(t\right)dB_{3}\left(t\right),\\ dR(t)= & \left[\left(\theta +\mu +u_{1}\left(t\right)\right)I_{1}\left(t\right)+\left(\theta +\mu +u_{2}\left(t\right)\right)I_{2}\left(t\right)+\gamma S\left(t\right)-dR\left(t\right)\right]dt\\ & +\sigma_{4}R\left(t\right)dB_{4}\left(t\right). \end{array}\right.$$

where the initial condition is $S\left(0\right)\geq 0,I_{i}\left(0\right)\geq 0,R\left(0\right)\geq 0,\;i=1,2$.

For the sake of convenience, consider the following variable

$$x\left(t\right)={\left[x_{1}\left(t\right),x_{2}\left(t\right),x_{3}\left(t\right),x_{4}\left(t\right)\right]}^{T}\overset{▵}{=}{\left[S\left(t\right),I_{1}\left(t\right),I_{2}\left(t\right),R\left(t\right)\right]}^{T}.$$

Then, system (12) can be expressed as dx(t)=f(x(t),u(t))dt+g(x(t))dB(t), where functions f(x(t),u(t)) and g(x(t)) are four-dimensional vectors with components

$$\left\{\begin{array}{cc} & f_{1}\left(x\left(t\right),u_{1}\left(t\right),u_{2}\left(t\right)\right)=\Lambda -\left(\alpha +\beta \right)\langle k\rangle S\left(t\right)\left(I_{1}\left(t\right)+I_{2}\left(t\right)\right)-\left(d+\gamma \right)S\left(t\right),\\ & f_{2}\left(x\left(t\right),u_{1}\left(t\right),u_{2}\left(t\right)\right)=\alpha \langle k\rangle S\left(t\right)\left(I_{1}\left(t\right)+I_{2}\left(t\right)\right)-\left(d+\theta +\mu +u_{1}\left(t\right)\right)I_{1}\left(t\right),\\ & f_{3}\left(x\left(t\right),u_{1}\left(t\right),u_{2}\left(t\right)\right)=\beta \langle k\rangle S\left(t\right)\left(I_{1}\left(t\right)+I_{2}\left(t\right)\right)-\left(d+\theta +\mu +u_{2}\left(t\right)\right)I_{2}\left(t\right),\\ & f_{4}\left(x\left(t\right),u_{1}\left(t\right),u_{2}\left(t\right)\right)=\left(\theta +\mu +u_{1}\left(t\right)\right)I_{1}\left(t\right)+\left(\theta +\mu +u_{2}\left(t\right)\right)I_{2}\left(t\right)+\gamma S\left(t\right)-dR\left(t\right),\\ & g_{1}\left(x\left(t\right)\right)=\sigma_{1}S\left(t\right),g_{2}\left(x\left(t\right)\right)=\sigma_{2}I_{1}\left(t\right),g_{3}\left(x\left(t\right)\right)=\sigma_{3}I_{2}\left(t\right),g_{4}\left(x\left(t\right)\right)=\sigma_{4}R\left(t\right). \end{array}\right.$$

Next, we need to illustrate that the minimum number of spreaders and the minimum cost of control measures are achieved simultaneously. Therefore, we put forward the following objective function:

(13) $$J\left(u_{1}\left(t\right),u_{2}\left(t\right)\right)=E\left[\int_{0}^{T}A_{1}I_{1}\left(t\right)+A_{2}I_{2}\left(t\right)+\frac{1}{2}B_{1}u_{1}^{2}\left(t\right)+\frac{1}{2}B_{2}u_{2}^{2}\left(t\right)dt\right],$$

where $A_{1}$, $A_{2}$, $B_{1}$, $B_{2}$ are positive weight coefficients between the spreaders and the control variables, and *T* represents the final moment when the control strategy ends. The control problem can be equivalently regarded as finding $u_{1}^{*}\left(t\right)$ and $u_{2}^{*}\left(t\right)$, such that $J\left(u_{1}^{*}\left(t\right),u_{2}^{*}\left(t\right)\right)\leq J\left(u_{1}\left(t\right),u_{2}\left(t\right)\right)$, $(u_{1}\left(t\right),u_{2}\left(t\right))\in U$, where the control set *U* is defined as

$$U=\left\{\left.u\left(t\right)=(u_{1}\left(t\right),u_{2}\left(t\right))\right|0\leq u_{1}\left(t\right)\leq 1,0\leq u_{2}\left(t\right)\leq 1,t\in \left[0,T\right]\right\}.$$

Define the function $L\left(x\left(t\right),u\left(t\right)\right)=A_{1}I_{1}\left(t\right)+A_{2}I_{2}\left(t\right)+\frac{1}{2}B_{1}u_{1}^{2}\left(t\right)+\frac{1}{2}B_{2}u_{2}^{2}\left(t\right)$. According to the Pontryagin minimum principle in the stochastic case [30], the Hamiltonian function is defined as

$$H\left(x\left(t\right),u\left(t\right),p\left(t\right),q\left(t\right)\right)=L\left(x\left(t\right),u\left(t\right)\right)+\langle f(x(t),u(t)),p(t)\rangle +\langle g(x(t)),q(t)\rangle ,$$

where $\langle \cdot ,\cdot \rangle$ represents the inner product in the Euclidean space, $p\left(t\right)=[p_{1}\left(t\right),p_{2}\left(t\right)$, $p_{3}\left(t\right),p_{4}{\left(t\right)]}^{T}$ and $q\left(t\right)={\left[q_{1}\left(t\right),q_{2}\left(t\right),q_{3}\left(t\right),q_{4}\left(t\right)\right]}^{T}$ are two adjoint variables. We can draw the following conclusions.

**Theorem** **4.** Under the optimal control $u^{*}\left(t\right)$, corresponding to the optimal trajectory $x^{*}\left(t\right)={\left[S^{*}\left(t\right),I_{1}^{*}\left(t\right),I_{2}^{*}\left(t\right),R^{*}\left(t\right)\right]}^{T}$ of the model (12), there exist adjoint variables $p_{i}\left(t\right),i=1,2,\ldots ,4$ satisfying the following adjoint equations

$$\left\{\begin{array}{cc} dp_{1}\left(t\right)= & [\left(p_{1}\left(t\right)-p_{2}\left(t\right)\right)\alpha \langle k\rangle S^{*}\left(t\right)\left(I_{1}^{*}\left(t\right)+I_{2}^{*}\left(t\right)\right)+\left(p_{1}\left(t\right)-p_{3}\left(t\right)\right)\beta \langle k\rangle S^{*}\left(t\right)(I_{1}^{*}\left(t\right)\\ & +I_{2}^{*}\left(t\right))+p_{1}\left(t\right)d+\left(p_{1}\left(t\right)+p_{4}\left(t\right)\right)\gamma -q_{1}\left(t\right)\sigma_{1}]dt+q_{1}\left(t\right)dB_{1}\left(t\right),\\ dp_{2}\left(t\right)= & [A_{1}+\left(p_{1}\left(t\right)-p_{2}\left(t\right)\right)\alpha \langle k\rangle S^{*}\left(t\right)+\left(p_{1}\left(t\right)-p_{3}\left(t\right)\right)\beta \langle k\rangle S^{*}\left(t\right)\\ & +\left(p_{2}\left(t\right)-p_{4}\left(t\right)\right)\left(\theta +\mu +u_{1}^{*}\left(t\right)\right)+p_{2}\left(t\right)d-q_{2}\left(t\right)\sigma_{2}]dt+q_{2}\left(t\right)dB_{2}\left(t\right),\\ dp_{3}\left(t\right)= & [A_{2}+\left(p_{1}\left(t\right)-p_{2}\left(t\right)\right)\alpha \langle k\rangle S^{*}\left(t\right)+\left(p_{1}\left(t\right)-p_{3}\left(t\right)\right)\beta \langle k\rangle S^{*}\left(t\right)\\ & +\left(p_{3}\left(t\right)-p_{4}\left(t\right)\right)\left(\theta +\mu +u_{2}^{*}\left(t\right)\right)+p_{3}\left(t\right)d-q_{3}\left(t\right)\sigma_{3}]dt+q_{3}\left(t\right)dB_{3}\left(t\right),\\ dp_{4}\left(t\right)= & (p_{4}\left(t\right)d-q_{4}\left(t\right)\sigma_{4})dt+q_{4}\left(t\right)dB_{4}\left(t\right), \end{array}\right.$$

*with the transversality conditions $x\left(0\right)=x_{0}$ and $p_{i}\left(T\right)=0,i=1,2,\ldots ,4$, the stochastic optimal control is*

$$\begin{array}{c} u_{1}^{*}\left(t\right)=\min\left\{\max\left\{\frac{\left(p_{2}\left(t\right)-p_{4}\left(t\right)\right)I_{1}^{*}\left(t\right)}{B_{1}},0\right\},1\right\},\\ u_{2}^{*}\left(t\right)=\min\left\{\max\left\{\frac{\left(p_{3}\left(t\right)-p_{4}\left(t\right)\right)I_{2}^{*}\left(t\right)}{B_{2}},0\right\},1\right\}. \end{array}$$

**Proof.**  The Hamiltonian function is expressed as

$$\begin{array}{cc} \; & H(x(t),u(t),p(t),q(t),t)\\ = & \;A_{1}I_{1}\left(t\right)+A_{2}I_{2}\left(t\right)+\frac{1}{2}B_{1}u_{1}^{2}\left(t\right)+\frac{1}{2}B_{2}u_{2}^{2}\left(t\right)\\ \; & +p_{1}\left(t\right)\left[\Lambda -\left(\alpha +\beta \right)\langle k\rangle S\left(t\right)\left(I_{1}\left(t\right)+I_{2}\left(t\right)\right)-\left(d+\gamma \right)S\left(t\right)\right]\\ \; & +p_{2}\left(t\right)\left[\alpha \langle k\rangle S\left(t\right)\left(I_{1}\left(t\right)+I_{2}\left(t\right)\right)-\left(d+\theta +\mu +u_{1}\left(t\right)\right)I_{1}\left(t\right)\right] \end{array}$$

$$\begin{array}{cc} \; & +p_{3}\left(t\right)\left[\beta \langle k\rangle S\left(t\right)\left(I_{1}\left(t\right)+I_{2}\left(t\right)\right)-\left(d+\theta +\mu +u_{2}\left(t\right)\right)I_{2}\left(t\right)\right]\\ \; & +p_{4}\left(t\right)\left[\left(\theta +\mu +u_{1}\left(t\right)\right)I_{1}\left(t\right)+\left(\theta +\mu +u_{2}\left(t\right)\right)I_{2}\left(t\right)+\gamma S\left(t\right)-dR\left(t\right)\right]\\ \; & +q_{1}\left(t\right)\sigma_{1}S\left(t\right)+q_{2}\left(t\right)\sigma_{2}I_{1}\left(t\right)+q_{3}\left(t\right)\sigma_{3}I_{2}\left(t\right)+q_{4}\left(t\right)\sigma_{4}R\left(t\right). \end{array}$$

According to the Pontryagin minimum principle in the stochastic case, we have $x^{*}\left(t\right)$, $u^{*}\left(t\right)$ satisfying

$$dx^{*}\left(t\right)=\frac{\partial H(x^{*}\left(t\right),u^{*}\left(t\right),p\left(t\right),q\left(t\right),t)}{\partial p(t)}dt+g\left(x^{*}\left(t\right)\right)dB\left(t\right),$$

$$dp\left(t\right)=-\frac{\partial H(x^{*}\left(t\right),u^{*}\left(t\right),p\left(t\right),q\left(t\right),t)}{\partial x(t)}dt+q\left(t\right)dB\left(t\right),$$

$$H\left(x^{*}\left(t\right),u^{*}\left(t\right),p\left(t\right),q\left(t\right),t\right)=\min_{u(t)\in U}H\left(x\left(t\right),u\left(t\right),p\left(t\right),q\left(t\right),t\right).$$

Therefore, the following adjoint equation can be obtained.

$$\left\{\begin{array}{cc} dp_{1}\left(t\right)= & \;[\left(p_{1}\left(t\right)-p_{2}\left(t\right)\right)\alpha \langle k\rangle S^{*}\left(t\right)\left(I_{1}^{*}\left(t\right)+I_{2}^{*}\left(t\right)\right)+\left(p_{1}\left(t\right)-p_{3}\left(t\right)\right)\beta \langle k\rangle S^{*}\left(t\right)(I_{1}^{*}\left(t\right)\\ & +I_{2}^{*}\left(t\right))+p_{1}\left(t\right)d+\left(p_{1}\left(t\right)+p_{4}\left(t\right)\right)\gamma -q_{1}\sigma_{1}]dt+q_{1}dB_{1}\left(t\right),\\ dp_{2}\left(t\right)= & \;[A_{1}+\left(p_{1}\left(t\right)-p_{2}\left(t\right)\right)\alpha \langle k\rangle S^{*}\left(t\right)+\left(p_{1}\left(t\right)-p_{3}\left(t\right)\right)\beta \langle k\rangle S^{*}\left(t\right)\\ & +\left(p_{2}\left(t\right)-p_{4}\left(t\right)\right)\left(\theta +\mu +u_{1}^{*}\left(t\right)\right)+p_{2}\left(t\right)d-q_{2}\left(t\right)\sigma_{2}]dt+q_{2}\left(t\right)dB_{2}\left(t\right),\\ dp_{3}\left(t\right)= & \;[A_{2}+\left(p_{1}\left(t\right)-p_{2}\left(t\right)\right)\alpha \langle k\rangle S^{*}\left(t\right)+\left(p_{1}\left(t\right)-p_{3}\left(t\right)\right)\beta \langle k\rangle S^{*}\left(t\right)\\ & +\left(p_{3}\left(t\right)-p_{4}\left(t\right)\right)\left(\theta +\mu +u_{2}^{*}\left(t\right)\right)+p_{3}\left(t\right)d-q_{3}\left(t\right)\sigma_{3}]dt+q_{3}\left(t\right)dB_{3}\left(t\right),\\ dp_{4}\left(t\right)= & \;(p_{4}\left(t\right)d-q_{4}\left(t\right)\sigma_{4})dt+q_{4}\left(t\right)dB_{4}\left(t\right). \end{array}\right.$$

Finally, the partial derivatives with respect to $u_{1}$ and $u_{2}$ are calculated for Hamiltonian functions, respectively. Combining with the range of the control set *U*, we obtain the following optimal control

$$\begin{array}{c} u_{1}^{*}\left(t\right)=\min\left\{\max\left\{\frac{\left(p_{2}\left(t\right)-p_{4}\left(t\right)\right)I_{1}^{*}\left(t\right)}{B_{1}},0\right\},1\right\},\\ u_{2}^{*}\left(t\right)=\min\left\{\max\left\{\frac{\left(p_{3}\left(t\right)-p_{4}\left(t\right)\right)I_{2}^{*}\left(t\right)}{B_{2}},0\right\},1\right\}. \end{array}$$

□

## 5. Numerical Simulation

In this part, we select different parameters (see Table 2) to conduct approximate simulations on the deterministic system and the stochastic system to strengthen and verify the models and dynamic analyses presented in the previous sections. We use the Milstein method [31] to carry out numerical simulations on the stochastic system (2) with a step size of $\Delta =2\times 10^{-2}$. To distinguish the influences of white noise and noise-free conditions on its asymptotic behavior, the sample trajectories of the deterministic and stochastic models are simulated.

### 5.1. Sensitivity Analysis

The rumor-spreading threshold $R_{0}$ is a key parameter in studying rumor dynamics. Recent studies, such as [32,33], have demonstrated the necessity of sensitivity analyses on $R_{0}$. Here, we adopt the normalized forward sensitivity index method [34]. This method assesses how the state variable *u* changes relative to a change in the parameter *v*, expressed as follows:

$$\pi_{v}^{u}:=\frac{\partial u}{\partial v}\times \frac{v}{u}.$$

**Figure 2:**
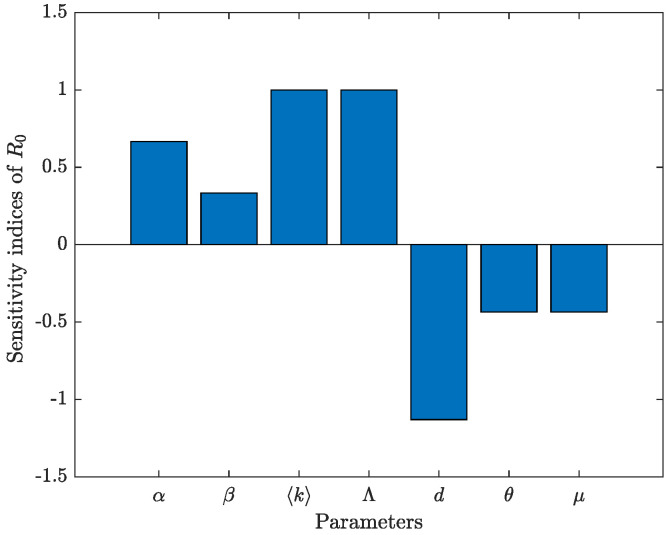
Bar chart of the sensitivity of $R_{0}$ to parameters with the value data 1.

As shown in Figure 2, the system is highly sensitive to the rates α and β at which the ignorant are transformed into rumor spreaders, the immigration rate Λ, and the average degree $\langle k\rangle$ of the network. Moreover, it is inversely sensitive to the forgetting rate θ in the spreader group and the punishment mechanism μ, and it is highly inversely sensitive to the removal rate *d* of users.

### 5.2. Asymptotic Behavior Simulation of Stochastic Model

**Figure 3:**
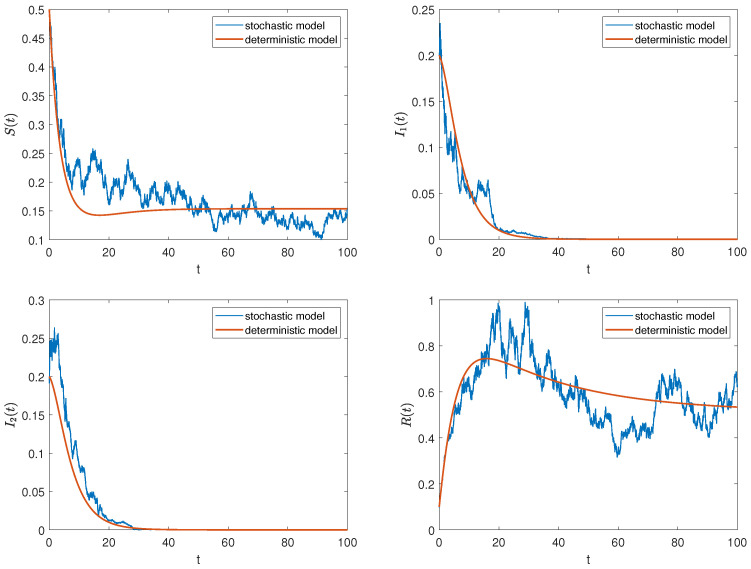
The trajectories of deterministic and stochastic systems for $R_{0}<1$.

Firstly, we consider the parameter values from data 1 and the initial value (0.5,0.2,0.2,0.1). At this time, $R_{0}=0.87<1$ and $\sigma_{1}=0.1,\sigma_{2}=0.2,\sigma_{3}=0.2,\sigma_{4}=0.1$ satisfy the conditions in Theorem 2, and all positive solutions of model (2) fluctuate around the rumor-free equilibrium point of model (1). As shown in Figure 3, the solution trajectories of the stochastic model fluctuate up and down around the solution trajectories of the deterministic model. As time goes by, the number of rumor spreaders gradually tends to 0, while the ignorants and the stiflers remain as the survivors in the system.

**Figure 4:**
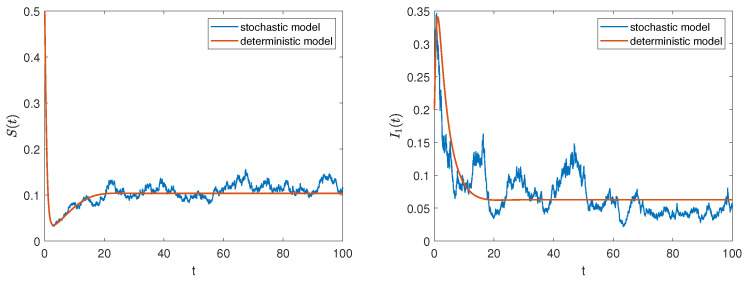

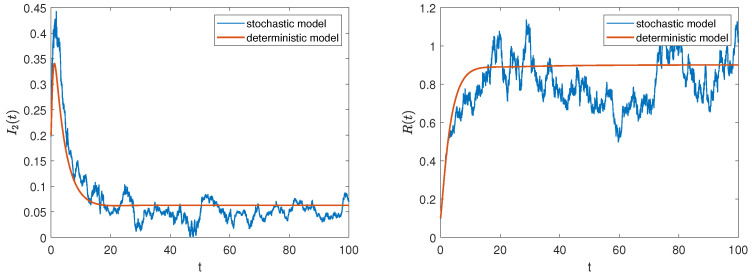
The trajectories of deterministic and stochastic systems for $R_{0}>1$.

In addition, we consider the parameter values from data 2 and the initial value (0.5,0.2,0.2,0.1). Then, $R_{0}=8.06>1$ and $\sigma_{1}=0.1$, $\sigma_{2}=0.2$, $\sigma_{3}=0.2$, $\sigma_{4}=0.1$. According to Theorem 3, all positive solutions of model (2) fluctuate around the rumor prevalence equilibrium of model (1). Similarly, the solution trajectories of the stochastic model in Figure 4 fluctuate around the solution trajectories of the deterministic model, which clearly supports this analytical result. Meanwhile, we find that when $R_{0}>1$, four types of groups coexist in the system and eventually tend to stabilize. However, once affected by external disturbances, this stable state will be broken, and the rumor will continue to spread.

**Figure 5:**
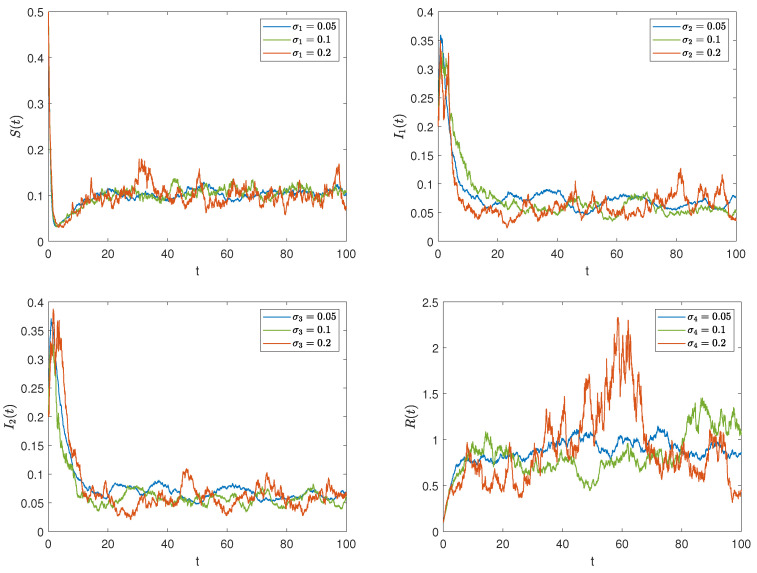
The impact of different interference intensities on rumor propagation.

On the basis of setting the parameter values as data 2 and the initial value as (0.5,0.2,0.2,0.1), we change the stochastic interference intensities to $\sigma_{i}=0.05,0.1,$ 0.2(i=1,2,3,4). Figure 5 depicts the evolutionary trajectories of the densities of each group in the system over time under different noise intensities. It can be seen that as the noise intensity increases, the amplitude of the stochastic vibration becomes larger, making the rumor more difficult to control. This verifies the changes we analyzed in Remark 1.

**Remark** **2.** *It can be observed from Figure 3 and Figure 4 that external stochastic disturbances lead to fluctuations in the solution trajectory of the stochastic model, causing it to oscillate around the steady states of the deterministic model, but it does not completely deviate from the deterministic trajectory. Furthermore, an increase in noise intensity complicates the control of rumors, as illustrated in Figure 5.*

### 5.3. Simulation and Comparison of Stochastic Optimal Control

**Figure 6:**
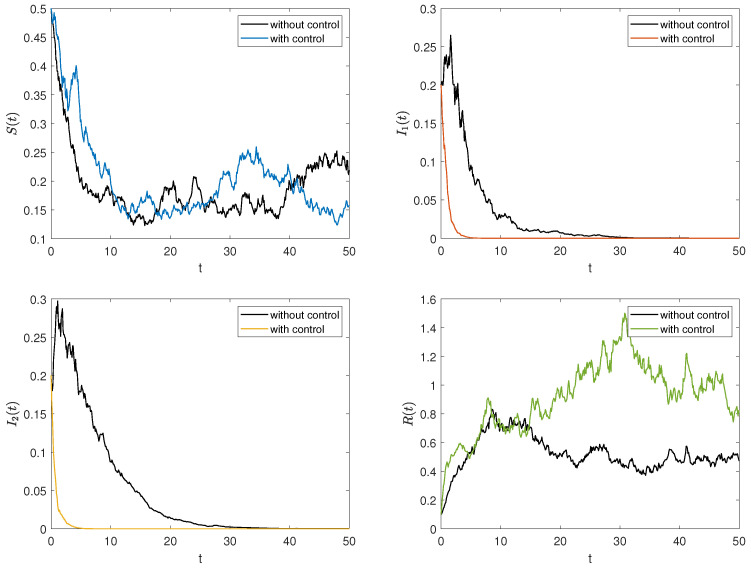
The trajectories of the stochastic system under optimal control.

Now, we combine the parameter data 1 in Table 2 to simulate stochastic systems with and without optimal control, examining how control affects the numbers of S(t), $I_{1}\left(t\right)$, $I_{2}\left(t\right)$, and R(t). The initial condition is (0.5,0.2,0.2,0.1), with $A_{1}=10$, $A_{2}=5$, $B_{1}=0.55$, $B_{2}=0.35$ in the objective function. Figure 6 illustrates the density changes of each group under both stochastic optimal control and no control. Results indicate that stochastic optimal control increases the densities of ignorants and stiflers while decreasing the density of spreaders, which tends to 0 more rapidly.

**Figure 7:**
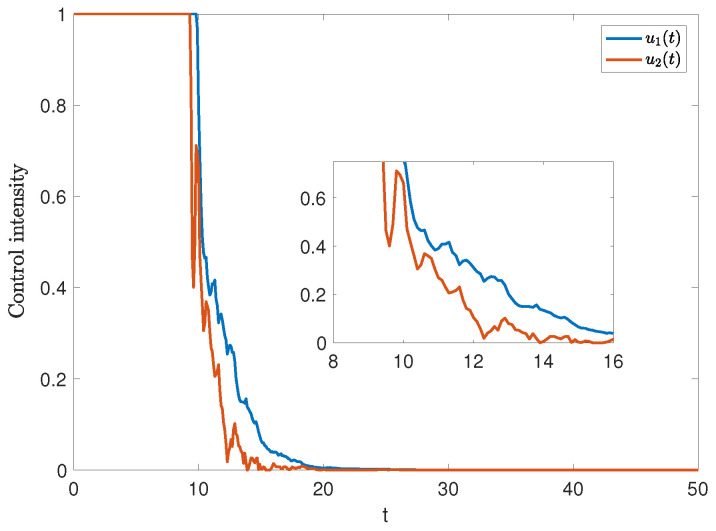
The trajectories of $u_{1}\left(t\right)$ and $u_{2}\left(t\right)$ under stochastic optimal control.

Next, Figure 7 further shows the temporal dynamics of stochastic optimal control intensity. Initially, the control variables reach their maximum values, followed by a rapid decline over time, ultimately approaching 0. This trend highlights the effectiveness of initial interventions.

**Remark** **3.** *The stochastic optimal control strategy effectively suppresses rumor spread and mitigates its negative impact (see Figure 6), particularly when intervention occurs early in the dissemination process (see Figure 7). In response, the government should strengthen oversight and establish a robust rumor-refuting mechanism. Additionally, media and relevant platforms must swiftly release accurate and transparent information to limit the spread of rumors.*

**Remark** **4.** *To further validate the cost-efficiency of the optimal control strategy, we compare the function $J(u_{1}\left(t\right),u_{2}\left(t\right))$ under two scenarios: with optimal control and without control. The results demonstrate that the cost under optimal control is significantly lower than that without control, despite the additional expenses associated with implementing the control measures. This is attributed to the substantial reduction in the number of spreaders $I_{1}\left(t\right)$ and $I_{2}\left(t\right)$, which outweighs the costs of the control measures.*

## 6. Conclusions

In this study, we have developed a multilingual stochastic S2IR rumor-spreading model. Meanwhile, we have investigated the existence and uniqueness of the global positive solution for the stochastic rumor model, as well as the asymptotic behavior of the solution near the steady states. Furthermore, we have proposed a stochastic optimal control strategy aimed at minimizing the number of rumor spreaders at the lowest possible cost, providing a theoretical reference for effectively controlling rumors.

In future work, we plan to collaborate with relevant organizations and utilize real-world data to further validate and refine our model, addressing practical problems in multilingual rumor control.

## Figures and Tables

**Table 1:** Definition of relevant parameters.

Parameters	Definition
〈k〉	Average degree of homogeneous network
Λ	The immigration rate of S(t) per unit time
*d*	The removal rate of users per unit time
α	The probability of S(t) contact $I_{i}\left(t\right)$ and become $I_{1}\left(t\right)$
β	The probability of S(t) contact $I_{i}\left(t\right)$ and become $I_{2}\left(t\right)$
θ	Spreaders’s forgetting rate
μ	Punishment mechanism for spreaders
γ	The probability of the ignorants transforming into the stiflers

**Table 2:** System parameters.

	Parameters	〈k〉	Λ	*d*	α	β	θ	μ	γ
Data									
data 1		5	0.02	0.03	0.04	0.02	0.1	0.1	0.1
data 2		5	0.05	0.05	0.3	0.2	0.12	0.14	0.12

## Data Availability

The original contributions presented in this study are included in the article. Further inquiries can be directed to the corresponding author.
